# Peri-Implant Surgical Treatment Downregulates the Expression of sTREM-1 and MMP-8 in Patients with Peri-Implantitis: A Prospective Study

**DOI:** 10.3390/ijerph19063627

**Published:** 2022-03-18

**Authors:** Glaucia Schuindt Teixeira Neves, Gayathiri Elangovan, Mayla Kezy Silva Teixeira, João Martins de Mello-Neto, Santosh Kumar Tadakamadla, Eduardo José Veras Lourenço, Daniel Moraes Telles, Carlos Marcelo Figueredo

**Affiliations:** 1 Department of Prosthodontics, School of Dentistry, Rio de Janeiro State University, Rio de Janeiro 20551-030, Brazil; nevesdds@gmail.com (G.S.T.N.); elourenco@br.inter.net (E.J.V.L.); daniel@telles.odo.br (D.M.T.); 2 Department of Endodontics, School of Dentistry, University of Texas, Houston, TX 77054, USA; 3 School of Medicine and Dentistry, Griffith University, Gold Coast, Queensland 4222, Australia; gayathiri.elangovan@griffithuni.edu.au (G.E.); j.martinsdemelloneto@griffith.edu.au (J.M.d.M.-N.); s.tadakamadla@griffith.edu.au (S.K.T.); c.dasilvafigueredo@griffith.edu.au (C.M.F.); 4 Menzies Health Institute Queensland, Queensland 4222, Australia; 5 Department of Periodontology, School of Dentistry, Rio de Janeiro State University, Rio de Janeiro 20551-030, Brazil

**Keywords:** sTREM-1, MMP-8, PGLYRP-1, peri-implant mucositis, peri-implantitis, peri-implant treatment

## Abstract

sTREM-1 and its ligand PGLYRP1 play an essential role in the inflammatory process around teeth and implants. In this study, we aimed to evaluate the impact of peri-implant treatment on the salivary levels of the sTREM-1/PGLYRP-1/MMP-8 axis after 3 months. A total of 42 participants (with a mean age of 61 years old ± 7.3) were enrolled in this longitudinal study, 24 having peri-implant mucositis (MU) and 18 having peri-implantitis (PI). Clinical peri-implant parameters, such as probing pocket depth (PPD), % of plaque, and bleeding on probing (BOP), and the whole unstimulated saliva samples were evaluated at baseline and 3 months after treatment. The MU group received nonsurgical peri-implant treatment, while the PI group received open-flap procedures. The levels of sTREM-1, PGLYRP-1, MMP-8, and TIMP-1 were analyzed using enzyme-linked immunosorbent assays. BOP, plaque levels, and PPD significantly reduced after treatment in both groups. A significant decrease in the salivary levels of sTREM-1, MMP-8, and TIMP-1 in the PI group and PGLYRP1 and TIMP-1 in the MU group were observed. Salivary levels of sTREM-1 were significantly reduced in patients with PI but not with MU. Additionally, peri-implant treatment had a significantly higher impact on MMP-8 reduction in patients with PI than in those with MU.

## 1. Introduction

Peri-implant diseases are characterized by inflammatory reactions in the tissues surrounding an osseointegrated implant and can be divided into two main types: peri-implant mucositis and peri-implantitis. Peri-implant mucositis (MU) is a reversible inflammation restricted to soft tissues. At the same time, peri-implantitis (PI) is characterized by progressive loss of supporting bone associated with an inflammatory reaction around an implant, resulting in implant loss [1,2,3]. A recent meta-analysis estimated the weighted mean prevalence of peri-implant mucositis and peri-implantitis at 43% (CI: 32–54%) and 22% (CI: 14–30%), respectively [4]. Moreover, Zhang et al. [5] reported that the prevalence of peri-implantitis at patient-level and implant level is slightly reduced in China, at 16% and 11.2%, respectively. According to Fu and Wang [6], treatment success was favorable in the short term, with 75% of the cases unresolved or recuring after 5 years.

Despite the associations and similarities regarding the clinical characteristics of peri-implantitis with periodontitis and mucositis with gingivitis, there are critical histopathological differences between the mentioned diseases. Both have a dense inflammatory infiltrate, but this is more pronounced in peri-implantitis [7]. The pathogenic pathway of peri-implant diseases shows that both mucositis and peri-implantitis result from an imbalance between the microbiota and host response, presenting a dense inflammatory infiltrate in the connective tissue, predominantly characterized by macrophages and polymorphonuclear leukocytes [7,8,9].

Triggering receptor expressed on myeloid cells 1 (TREM-1) is a surface molecule of the immunoglobulin superfamily, identified as an important modulator of the inflammatory response. Cells such as monocytes, macrophages, and neutrophils express TREM-1 during inflammatory and infectious processes [10,11]. The synergism between TREM-1 and pattern recognition receptors amplifies the inflammatory response, positively regulating the production of pro-inflammatory cytokines [10,12]. sTREM-1, the soluble form of TREM-1, results from the membrane-bound cleavage catalyzed by the presence of matrix metalloproteases (MMP) [13,14]. sTREM-1 levels have been found in higher saliva and gingival crevicular fluid of patients with periodontitis [15,16,17,18]. Bostanci et al. [19] have shown that TREM-1 regulates the IL-17A-RANKL/OPG axis and bone loss in experimental periodontitis, suggesting that TREM-1 regulation might have a potential therapeutic role in the treatment of human periodontitis. Our group has demonstrated that sTREM-1 and its physiological ligand, the peptidoglycan recognition protein (PGLYRP1), can be detected in the saliva of patients with the peri-implant disease [20]. Still, no study has addressed the impact of peri-implant treatment on the sTREM-1/PGLYRP-1/MMP-8 axis.

PGLYRP1 is a protein with bactericidal activity for Gram-positive and Gram-negative bacteria found in tertiary neutrophil granules [21,22] and, after binding to TREM-1, it increases neutrophil and macrophage cytokine production [23]. Increased salivary levels of sTREM-1 and PGLYRP1 were found in patients with chronic kidney disease and poor oral health and were positively correlated with patients with deeper probing depth (minimum of two sites with ≥6 mm) [24]. Therefore, we hypothesized that peri-implant treatment could prevent sTREM-1 binding to PGLYRP1 by reducing MMP-8 levels.

The treatment of peri-implant diseases aims to re-establish the original tissue health condition around dental implants. Peri-implant mucositis can be treated effectively using nonsurgical treatment—professional supragingival instrumentation, laser treatments, photodynamic therapy, or even local or systemic antibiotics [25]. On the other hand, the nonsurgical treatment of peri-implantitis is unpredictable [26], and surgical treatment seems to present a better resolvability of peri-implantitis if used through a sequence of therapeutic procedures that increase the potential of disinfection of the lesion [27]. Although biofilm elimination from the implant surface is the primary objective of the peri-implant treatment, peri-implant treatment has also been used to modulate the expression of inflammatory biomarkers [28]. Both surgical and nonsurgical treatment modalities have the potential to significantly reduce the levels of pro-inflammatory cytokines, such as IL-1β, IL-6, MMP-8, and TNF-α [29,30,31].

Previously, our group showed that clinical parameters and salivary levels of CSF-1 and S100A8/A9 can be improved by nonsurgical peri-implant treatment in peri-implant mucositis patients, and surgical in peri-implantitis patients in three-month follow-ups [32]. Herein, we aimed to evaluate the impact of peri-implant treatment in the salivary levels of the sTREM-1/PGLYRP1/MMP-8 axis at a three-month follow-up. Our null hypothesis was that peri-implant treatment had no impact on the salivary expression of sTREM-1, PGLYRP1, MMP-8 and TIMP1.

## 2. Materials and Methods

### 2.1. Study Population

A total of 42 participants with osseointegrated implants (25 males and 17 females, mean age of 61 years old ± 7.3) were enrolled in this longitudinal study, 24 having peri-implant mucositis, and 18 having peri-implantitis. To be considered as osseointegrated, we used the following criteria: clinically stable implant; without mobility; radiographic evaluation without any evidence of radiolucency; and the absence of persistent and/or irreversible signs and symptoms, such as pain, infections, neuropathies, and paresthesia [33]. To be included, patients were partially edentulous with two or more implant-retained prostheses (regardless of their intraoral location or commercial brand) in function for at least six months. Each patient had a minimum of 2 inflamed implants. The implants presented an external hexagon, morse taper, or internal hexagon systems (Neodent^®^, Curitiba, Brazil or SIN^®^, São Paulo, Brazil). The exclusion criteria were smokers, pregnant, breastfeeding women, and patients with chronic diseases (such as chronical kidney disease or diabetes). Patients who had received periodontal or peri-implant therapy in the preceding six months or had used medication, such as antibiotics and anti-inflammatory drugs, were also excluded. The study protocol was approved by the ethics committee of University Hospital Pedro Ernesto—Rio de Janeiro State University—UERJ, Rio de Janeiro, Brazil (HUPE—UERJ) (REF: 20220219.4.0000.5259). All patients signed written informed consent per the Declaration of Helsinki.

All patients underwent a complete periapical radiographic examination to aid in diagnosis. The patients were instructed not to eat or drink for 1 h before the appointment. The sample collection and clinical examinations were performed by two calibrated dentists (G.S.T. and M.K.S.T.) using a periodontal probe (PCR 15, Hu-Friedy, Chicago, IL, USA) in six sites per implant. The inter-examiner calibration was performed by examining five patients twice. The following parameters were registered for calibration: probing pocket depth (PPD), bleeding on probing (BOP), and plaque index. The agreement for PD measurements was 95.6% within ±1 mm. In the present study, all the evaluated dental implants were bone level and only restored after a clinical and radiographic evaluation showed that no implant thread was exposed.

Patients with clinically inflamed sites, bleeding on gentle probing, and without any significant radiographic bone loss (bone loss around the implant not reaching the first thread) were allocated to the MU group. Patients with inflamed sites (presence of bleeding and/or suppuration on gentle probing), increased probing depths and/or recession of the mucosal margin, and bone loss involving at least two implant threads were allocated to the PI group [1,26]. All patients enrolled in the present study had developed biofilm-induced inflammation. Any bone loss resulting from iatrogenic trauma or other factors not related to biofilm-induced inflammation were not included.

### 2.2. Peri-Implant Treatment

The patients with peri-implant mucositis received nonsurgical treatment, which included oral hygiene instructions, professional supragingival scaling, dental/implant cleaning using rubber cups, polishing paste, and using a bicarbonate jet (Jetflex I—Dentflex, São Paulo, Brazil). PI patients were submitted to the same protocol plus surgical procedures. Peri-implant treatment was performed by an experienced periodontist (EJVL). After local anesthesia, intrasulcular incision and elevation of a mucoperiosteal flap were executed to access implant threads and bone defects. Granulation tissue and biofilm were removed with Teflon hand instruments (Implacare—Hu-Friedy^®^, Chicago, IL, USA). The implants were polished using a rubber cup with non-abrasive polishing paste and bicarbonate jet (Jetflex I—Dentflex, São Paulo, Brazil), and washed with a sterile physiological saline solution. The region was rinsed with a 2% chlorhexidine solution, and flaps were sutured. Postoperative instructions and drug prescriptions were given, including antibiotics (500 mg amoxicillin every 8 h for seven days) and anti-inflammatory (nimesulide 100 mg every 12 h for five days). The simple sutures were used with 5.0 nylon thread and removed after 15 days. For both MU and PI patients, nonsurgical periodontal treatment was also performed when needed. Oral hygiene instruction was reinforced. Clinical examinations and sample collections were carried out at baseline and three months after treatment.

### 2.3. Saliva Collection

Unstimulated saliva was collected from all patients. Patients refrained from eating and drinking for at least 1 h before saliva samples were collected. Five minutes after rinsing their mouth with tap water, participants expectorated at least 1 mL of non-stimulated saliva into an Eppendorf containing 20 μL of protease inhibitor (Sigma-Aldrich, St. Louis, MO, USA). Samples were then centrifuged at 8000 rpm for 5 min, and the supernatant was collected and stored at −80 °C until analysis.

### 2.4. sTREM-1, PGLYRP1, MMP-8, and TIMP-1 Immunoassays

According to the manufacturer’s instructions, the levels of sTREM-1, PGLYRP1, MMP-8, and TIMP-1 were determined using commercial enzyme-linked immunosorbent assays (R&D Systems, Minneapolis, MN, USA), with sTREM-1 with a dilution factor of 1:2 and PGLYRP1, MMP-8, and TIMP-1 with a dilution factor of 1:100. Readings were made using a microplate spectrophotometer with a wavelength set at 450 nm (Thermo Scientific™, Multiskan Sky Microplate Reader™, Massachusetts, EUA). Samples below the limit of detection were set as 0.

### 2.5. Statistical Analysis

Statistical analyses were performed using the Statistical Package for Social Sciences (SPSS), version 24 (IBM Corporation, Armonk, NY, USA). The Shapiro–Wilk test evaluated data normality. The continuous variables are presented as mean and standard deviation (SD) or median and interquartile range. Categorical variables are presented as frequencies. The Mann–Whitney test was used to compare the continuous variables between the MU and PI groups at baseline and three-month follow-ups. The Wilcoxon signed-rank test was performed for paired samples. The difference between before and after treatment was calculated and presented as Delta variation (Δ). Statistical significance was set at *p* ≤ 0.05. The correlations were assessed using the Spearman correlation coefficient. The correlation values for variables to be considered relevant were R ≥ 0.5 and *p* ≤ 0.01.

## 3. Results

### 3.1. Demographics and Clinical Parameters

There were no significant differences between the groups regarding age (MU group: 63.38 ± 8.21 and PI group: 61.00 ± 7.12, *p* = 0.366) and gender (MU group: 14 males and 10 females; PI group: 11 males and 7 females, *p* = 0.689). The average number of implants per patient for the MU and PI groups was 4.79 ± 1.86 and 6.38 ± 2.74 (*p *= 0.171), respectively.

At baseline, peri-implantitis patients presented a significantly higher probing pocket depth than that of patients with peri-implant mucositis (*p* < 0.001) (Table 1). No significant differences were found for % plaque (*p* = 0.726) and % BOP (*p* = 0.489) between the groups (Table 1). Three months after peri-implant treatment, both groups showed significant improvements in all clinical parameters evaluated (Table 1).

### 3.2. Salivary Parameters

In the MU group, the cytokine detectability of sTREM-1, PGLYRP1, MMP-8, and TIMP-1 was 79%, 100%, 83%, and 96% at baseline, and 71%, 100%, 79%, and 87% after treatment, respectively. In the PI group, the cytokine detectability of sTREM-1, PGLYRP-1, MMP8, and TIMP-1 was 89%, 94%, 100%, and 100% at baseline, and 72%, 89%, 61%, and 100% after treatment, respectively.

**Figure 1:**
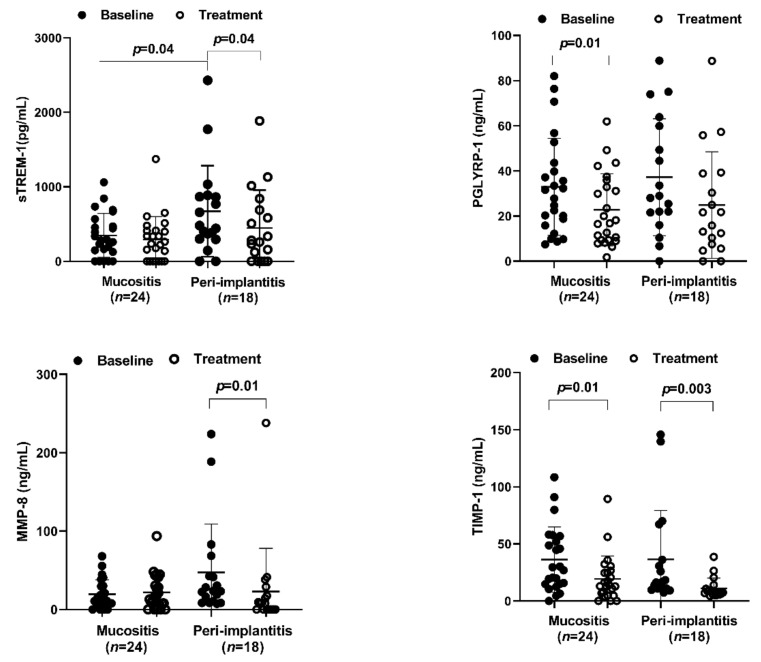
Levels of sTREM-1, PGLYRP-1, MMP-8, and TIMP-1 in saliva from individuals with peri-implant mucositis and peri-implantitis before and after treatment. *p* ≤ 0.05 (Wilcoxon signed-rank test).

At baseline, patients with PI presented a significantly higher level of sTREM-1 (461.15; IQR = 547.35) than that of patients with MU (284.3; IQR = 411.57) (*p* = 0.04) (Figure 1). There was no significant difference between the groups for the other cytokines evaluated in saliva.

Three months after treatment, there was a significant decrease in the salivary levels of PGLYRP1 (*p* = 0.01) and TIMP-1 (*p* = 0.01) in patients with MU, but sTREM-1 (*p* = 0.247) and MMP-8 (*p* = 0.886) did not show significant differences (Figure 1). In the PI group, there was a significant decrease in the salivary levels of sTREM-1 (*p* = 0.04), MMP-8 (*p* = 0.01), and TIMP-1 (*p* = 0.003), but not in PGLYRP1 (*p* = 0.396) (Figure 1).

#### 3.2.1. The Variation between before and after (Delta)

**Figure 2:**
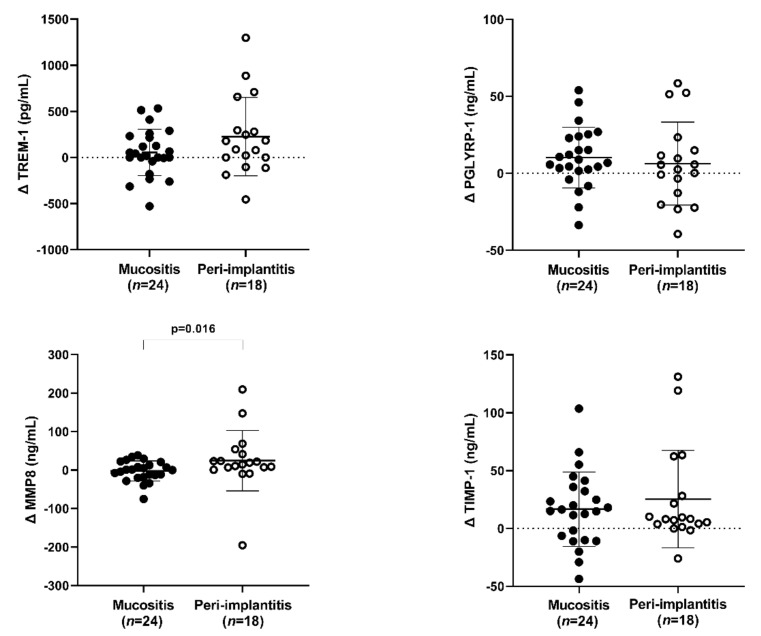
The treatment effect variation in salivary levels of sTREM-1, PGLYRP1, MMP-8, and TIMP-1 from peri-implant mucositis patients and peri-implantitis expressed in Δ. *p* ≤ 0.05 (Mann–Whitney U test).

The Delta variation analyses showed a significantly higher MMP-8 reduction in PI than in MU (*p* = 0.016), but it did not happen with sTREM-1 (*p* = 0.212), PGLYRP1 (*p* = 0.347), and TIMP-1 (*p* = 0.959) (Figure 2). 

#### 3.2.2. The Ratio Analysis

**Figure 3:**
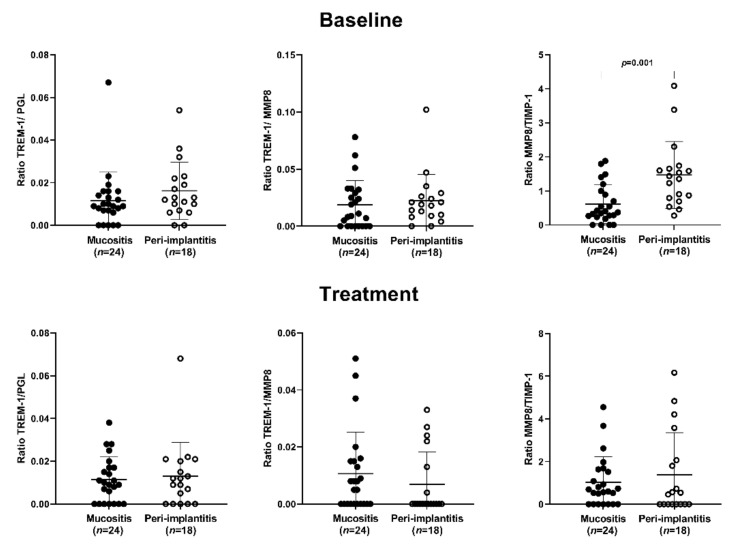
Ratio comparisons of total sTREM-1/PGLYRP-1, sTREM-1/MMP-8, and MMP-8/TIMP-1 in peri-implant mucositis patients and peri-implantitis patients at baseline and after peri-implant treatment. *p* ≤ 0.05 (Mann–Whitney U test).

**Figure 4:**
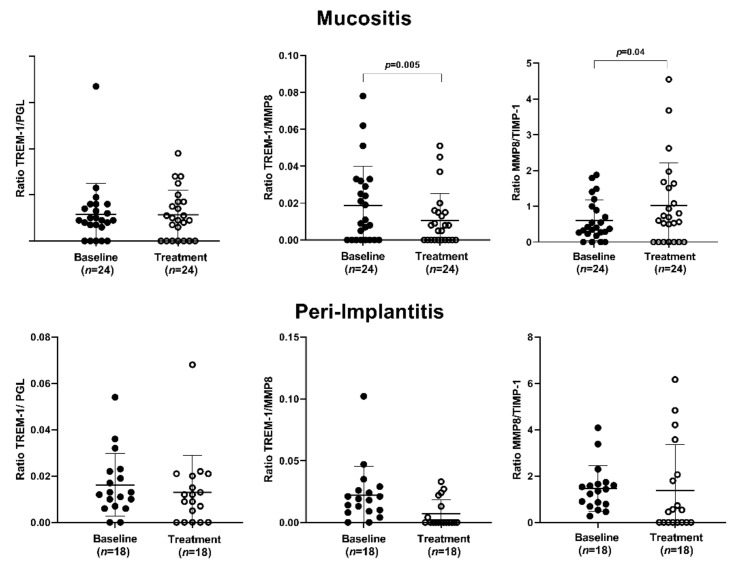
Peri-implant treatment effects on the ratio of total sTREM-1/PGLYRP-1, sTREM-1/MMP-8, and MMP-8/TIMP-1 in peri-implant mucositis patients and peri-implantitis patients before and after peri-implant treatment. *p* ≤ 0.05 (Wilcoxon signed-rank test).

The PI group presented a significantly higher MMP-8/TIMP-1 ratio than the MU group at baseline (*p* = 0.001) (Figure 3). Peri-implant treatment significantly reduced the sTREM-1/MMP-8 ratio (*p* = 0.05) and increased the MMP-8/TIMP-1 ratio (*p* = 0.04) in the MU group (Figure 4). No significant difference was observed in the PI group (Figure 4).

#### 3.2.3. Correlation Analysis

**Figure 5:**
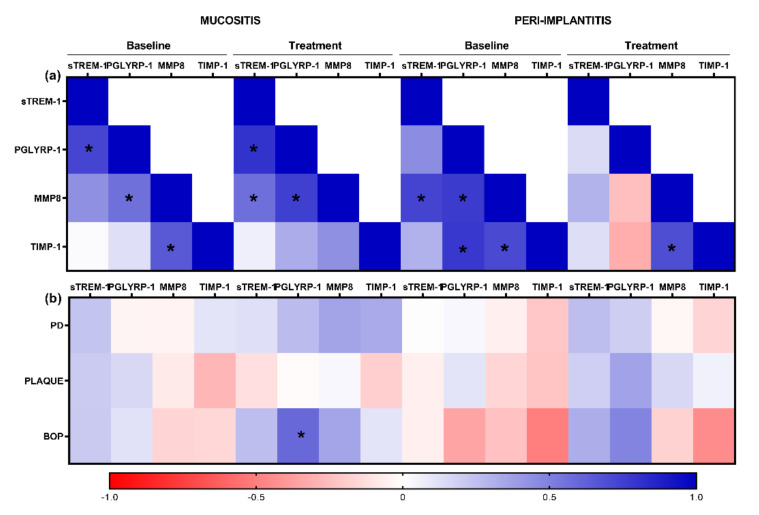
Heat maps exhibit correlations between the biomarkers and clinical parameters (**a**) and biomarkers (**b**) in saliva. * R ≥ 0.6, *p* ≤ 0.01 (Spearman correlation). PD: probing depth; BOP: bleeding on probing.

The salivary levels of PGLYRP1 had a significant positive correlation with bleeding on probing (R = 0.6, *p* = 0.002) after treatment in the MU group. There was no other significant correlation between the other cytokines levels and clinical parameters (Figure 5). 

## 4. Discussion

The present study demonstrated that salivary levels of sTREM-1, MMP-8, and TIMP-1 significantly decreased three months after peri-implant treatment in patients with PI. Those reductions were also associated with a significant reduction in bleeding on probing, plaque levels, and pocket depth.

A significant decrease in MMP-8 levels was observed in PI patients. In line with our studies, Bhavsar et al. [29] also demonstrated a significant reduction in MMP-8 concentration in the peri-implant crevicular fluid after three months of surgical treatment. On the other hand, Hentenaar et al. [34] did not find any MMP-8 reduction after nonsurgical therapy. The reason for this discrepancy is unknown, but the use of a nonsurgical approach, which is not the best option for peri-implantitis treatment [25], might have jeopardized their results. In our findings, there was no significant difference between both groups in baseline analysis, as shown in Figure 1. This is in line with the study conducted by Ziebolz et al. [35] that revealed no significant difference in MMP-8 among healthy mucositis and peri-implantitis sites. MMP-8 has been described as a potential adjunctive biomarker in peri-implant diseases [36]. It also has been suggested that polymorphism in the promoter region of the MMP-8 gene is associated with early bone loss [37]. In the present study, the findings support accumulating evidence that a reduction in salivary levels of MMP-8 was followed by notable improvements in clinical parameters. Ramseier et al. (2016) also found increased levels of MMP-8 in peri-implant fluid in the presence of inflammation around implants [38]. Since MMP-8 cleaves membrane-anchored TREM-1 [13], its reduction can help to decrease sTREM-1 levels, which might indirectly affect the local production of pro-inflammatory cytokines [12].

Along with MMP-8, a significant reduction in salivary levels of sTREM-1 after treatment was observed. This is the first paper to report that the expression of sTREM-1 can be downregulated by clinical intervention in patients with PI. Although the literature is scarce regarding the evaluation of sTREM-1 modulation after peri-implant treatment, our results align with those of Dubar et al. [18], who showed a reduction in sTREM-1 concentrations in the gingival crevicular fluid after scaling and root planning in patients with periodontitis. TREM-1 has an essential role in systemic inflammatory conditions, including cardiovascular disorders, obesity, sepsis, and pneumonia. According to Rudick et al. [39], methodological studies that manipulate a soluble TREM-1 (sTREM-1) could provide novel treatment possibilities for periodontal diseases. The use of TREM receptors as interventional tools to reverse or arrest progression of periodontal diseases and systemic infections could potentially be a goal to approach in therapy in the future [39].

Assuming that sTREM-1 is generated through the proteolytic cleavage of mature cell- surface-anchored TREM-1 by MMP [13,14], the reduction in MMP-8 might have reduced the amount of available sTREM in the intracellular area. Since it is known that MMP-8 is related to bone loss [40,41], we speculate that is the reason why the reduction in MMP-8 had a direct impact on sTREM levels in the PI group but not in the MU group. While TREM-1 is an important modulator of the inflammatory response and is also shed by the membranes of activated phagocytes being found in its soluble form (sTREM-1) in saliva and gingival crevicular fluid, the reduction in the expression of sTREM-1 has clinical applications to the pathogenesis of the peri-implant disease. sTREM-1 has the capacity to upregulate proinflammatory signaling and, consequently, cause tissue destruction. Its reduction might help the healing process by reducing myeloid cell activation in the cellular infiltrate formed in the peri-implant lesion.

PGLYRP1, the sTREM ligand, was also reduced after therapy. Considering its involvement in bactericidal activity for both Gram-positive and Gram-negative bacteria [22], it can be assumed that the significant reduction in % of plaque after treatment was reflected in both the salivary biomarkers and clinical inflammatory indexes. In our mucositis group, a positive correlation was observed between the expression of PGLYRP1 and bleeding on probing in the follow-up. Silbereisen et al. [42] also demonstrated a reduction in PGLYRP1 salivary levels after gingivitis treatment. A positive association between gingival inflammation and higher levels of PGLYRP1 was also observed. Thus, the present data indicated a reduction in sTREM and PGLYRP1 followed by a reduction in % of plaque and a significant reduction in mean probing pocket. It has been previously shown that higher levels of sTREM-1 and PGLYRP1 are associated with poor oral health and a higher inflammatory burden [24]. Furthermore, there was a strong positive correlation between sTREM-1 and PGLYRP1 in the mucositis group at baseline and after treatment, which is expected once the latter is a functional ligand of the first [23]. Such correlation also happened between PGLYRP1 and MMP-8 in the mucositis peri-implantitis groups.

The salivary levels of TIMP-1 significantly decreased after treatment in both groups. In line with our findings, the MMP-8/TIMP-1 axis ratio in saliva has been positively associated with the periodontal inflammatory burden index [43], which suggests that the reduction in salivary levels of the MMP-8/TIMP-1 axis reflected the inflammatory status of the disease. To date, no study has evaluated the impact of peri-implant treatment on TIMP-1 levels. Regarding periodontitis, Gorska et al. (2006) did not find any changes in the salivary TIMP-1 concentrations in periodontitis patients after scaling and root planning [44]. Fenol et al. [45] reported that TIMP-1 levels decreased after nonsurgical periodontal therapy. Marcaccini et al. [46] found a significant reduction in TIMP-2 but no difference for TIMP-1 three months after nonsurgical therapy. On the other hand, İnce et al. [47] found increased TIMP-1 levels after initial periodontal therapy associated with *Lactobacillus reuteri* containing probiotic supplementation in patients with chronic periodontitis. Such discrepancy is hard to explain, but as TIMPs have a critical role in indirectly remodeling the extracellular matrix, MMP–TIMP balance is critical to normal extracellular matrix function. A disruption in this balance has been implicated in numerous diseases [48]. Thus, the potential of MMP inhibition as a therapeutic target has been extensively explored.

Our clinical findings showed a significant reduction in pocket depth, % of plaque, and bleeding on probing in both the PI and the MU groups, which is in line with the literature [31]. Barootchi et al. [49] recently published a systematic review and meta-analysis reporting that conventional nonsurgical mechanical therapy alone is the standard treatment for MU. There is a lack of evidence supporting additional chemical/mechanical agents. Roccuzzo et al. [50] also reported high implant-level survival in the medium to long term when peri-implantitis therapy is followed by regular supportive care. The authors reported clinical improvements and stable peri-implant bone levels in most patients.

In this study, we used saliva collection. It presents advantages such as ease of manipulation, simple collection technique, and, in general, an adequate quantity to assess the potential of biomarkers in diagnosing and monitoring periodontal and peri-implant diseases [51,52,53]. Still, the salivary cytokines are connected with oral inflammation, making them potential biomarkers for disease detection [54]. Thus, saliva collection represents a more accessible means for clinical use by dentists. On the other hand, fluid collection is time consuming and technically demanding.

Caution is needed to interpret our results due to its limitations. Larger cohorts, more samples, and longer follow-ups would provide more robust information about the modulation of the TREM-1/PGLYRP1/MMP-8/TIMP-1 axis in the pathogenesis of peri-implant diseases and the impact of treatment on it.

## 5. Conclusions

In conclusion, our study showed a significant improvement in clinical parameters followed by a significant decrease in the salivary levels of sTREM-1 and MMP-8 in patients with PI but not in patients with MU three months after peri-implant treatment. Additionally, peri-implant treatment had a significantly higher impact on MMP-8 reduction in patients with PI than in those with peri-implant mucositis.

## Figures and Tables

**Table 1:** Clinical parameters of the study groups.

	Mucositis (*n* = 24)				Peri-Implantitis (*n* = 18)			
Variable	Baseline	*p*1	3 Months	*p*2	Baseline	*p*3	3 Months	*p*4
Peri-implant parameters								
Mean PPD	3.8 (0.7)	0.002	3.1 (0.9)	<0.001	5.04 (1.0)	0.005	4.0 (1.2)	0.016
% Plaque	74.3 (33.0)	0.019	49.1 (36.6)	0.726	70.3(33.1)	0.017	35.1 (47.8)	0.154
% BOP	88.2 (21.7)	0.001	47 (38.9)	0.489	93.44 (15.4)	0.004	49.0 (42.5)	0.906
Full mouth periodontal parameters								
Mean PPD	2.3 (0.3)	0.001	2.1 (0.3)	0.006	2.6 (0.3)	<0.001	2.3 (0.3)	0.021
Mean CAL	1.5 (1.0)	0.617	1.5 (0.9)	0.431	1.6 (0.8)	0.571	1.6 (0.8)	0.263
% Plaque	80.2(16.3)	0.010	69.2 (20.5)	0.060	68.6 (19.7)	0.218	61.4 (27.1)	0.237
% BOP	52 (22.6)	<0.001	35.4 (21.9)	0.303	58.2 (21.8)	0.002	34.6 (20.8)	0.970

PPD: probing pocket depth; CAL: clinical attachment level; BOP: bleeding on probing. P1: comparisons between baseline and 3 months after treatment in patients with mucositis. P2: comparisons between mucositis patients and peri-implantitis patients at baseline. P3: comparisons between baseline and 3 months after treatment in patients with peri-implantitis. P4: comparisons between mucositis patients and peri-implantitis patients after 3 months of treatment.

## Data Availability

Not applicable.
